# Supplementary material for: Impact of emergency department overcrowding on the occurrence of in-hospital cardiac arrest
Source: PLoS One. 2025 Jan 17;20(1):e0317457. doi: 10.1371/journal.pone.0317457 (PMC11741635; doi:10.1371/journal.pone.0317457)
Supplement: S1 Table — (DOCX) [file pone.0317457.s001.docx]

| **S1 Table. Characteristics of patients in the full study cohort and the propensity score-matched cohort, stratified by emergency department overcrowding, based on the number of total occupying patients above 80%** | | | | | | | | | | |
| --- | --- | --- | --- | --- | --- | --- | --- | --- | --- | --- |
| **Variables** | | **Full-study cohort** | | | | **Propensity score-matched cohort** | | | | |
|  |  | Overcrowding (n = 36200) | Non-overcrowding (n = 117153) | SMD | p-value | Overcrowding (n = 36200) | Non-overcrowding (n = 36200) | SMD | p-value | |
| Age | -39 | 10383 (28.68) | 40151 (34.27) | -0.1236 | <0.0001 | 10383 (28.68) | 10590 (29.25) | -0.0126 | 0.0548 | |
|  | 40-64 | 13211 (36.49) | 41672 (35.57) | 0.0192 |  | 13211 (36.49) | 13262 (36.64) | -0.0029 |  | |
|  | 65-79 | 9208 (25.44) | 25913 (22.12) | 0.0762 |  | 9208 (25.44) | 9135 (25.24) | 0.0046 |  | |
|  | 80- | 3398 (9.39) | 9417 (8.04) | 0.0462 |  | 3398 (9.39) | 3213 (8.88) | 0.0175 |  | |
| Male |  | 17083 (47.19) | 54113 (46.19) | 0.0200 | 0.0008 | 17083 (47.19) | 16993 (46.94) | 0.0050 | 0.5028 | |
| Emergency medical services |  | 7901 (21.83) | 29628 (25.29) | -0.0839 | <0.0001 | 7901 (21.83) | 7738 (21.38) | 0.0109 | 0.1410 | |
| Transfer in |  | 6696 (18.5) | 12647 (10.8) | 0.1984 | <0.0001 | 6696 (18.50) | 6401 (17.68) | 0.0210 | 0.0044 | |
| KTAS | 1 | 399 (1.10) | 1242 (1.06) | 0.0040 | <0.0001 | 399 (1.10) | 363 (1.00) | 0.0095 | 0.5020 | |
|  | 2 | 3161 (8.73) | 9627 (8.22) | 0.0182 |  | 3161 (8.73) | 3242 (8.96) | -0.0079 |  | |
|  | 3 | 10071 (27.82) | 28630 (24.44) | 0.0755 |  | 10071 (27.82) | 10120 (27.96) | -0.0030 |  | |
|  | 4 | 18387 (50.79) | 61702 (52.67) | -0.0375 |  | 18387 (50.79) | 18265 (50.46) | 0.0067 |  | |
|  | 5 | 4182 (11.55) | 15952 (13.62) | -0.0646 |  | 4182 (11.55) | 4210 (11.63) | -0.0024 |  | |
| Non-medical |  | 5296 (14.63) | 21564 (18.41) | -0.1069 | <0.0001 | 5296 (14.63) | 5471 (15.11) | -0.0137 | 0.0676 | |
| Chief complaints | Gastrointestinal | 7200 (19.89) | 23736 (20.26) | -0.0093 | <0.0001 | 7200 (19.89) | 7082 (19.56) | 0.0082 | 0.3391 | |
|  | General | 6242 (17.24) | 18772 (16.02) | 0.0323 |  | 6242 (17.24) | 6103 (16.86) | 0.0102 |  | |
|  | Neurological | 5576 (15.40) | 16858 (14.39) | 0.0281 |  | 5576 (15.40) | 5789 (15.99) | -0.0163 |  | |
|  | Cardiovascular | 3905 (10.79) | 11075 (9.45) | 0.0430 |  | 3905 (10.79) | 3912 (10.81) | -0.0006 |  | |
|  | Musculoskeletal | 3273 (9.04) | 10942 (9.34) | -0.0104 |  | 3273 (9.04) | 3291 (9.09) | -0.0017 |  | |
|  | Respiratory | 3235 (8.94) | 8083 (6.90) | 0.0714 |  | 3235 (8.94) | 3159 (8.73) | 0.0074 |  | |
|  | Skin | 2003 (5.53) | 8569 (7.31) | -0.0779 |  | 2003 (5.53) | 2080 (5.75) | -0.0093 |  | |
|  | ENT | 1698 (4.69) | 7631 (6.51) | -0.0862 |  | 1698 (4.69) | 1716 (4.74) | -0.0024 |  | |
|  | Others | 3068 (8.48) | 11487 (9.81) | -0.0478 |  | 3068 (8.48) | 3068 (8.48) | 0.0000 |  | |
| Severe disease |  | 4520 (12.49) | 12512 (10.68) | 0.0546 | <0.0001 | 4520 (12.49) | 4307 (11.90) | 0.0178 | 0.0155 | |
| Area | Monitoring area | 3159 (8.73) | 8992 (7.68) | 0.0372 | <0.0001 | 3159 (8.73) | 3155 (8.72) | 0.0004 | 0.8873 | |
|  | Bed area | 5420 (14.97) | 23137 (19.75) | -0.1339 |  | 5420 (14.97) | 5491 (15.17) | -0.0055 |  | |
|  | Chair area | 1515 (4.19) | 29386 (25.08) | -1.0436 |  | 1515 (4.19) | 1495 (4.13) | 0.0028 |  | |
|  | Fast track | 26106 (72.12) | 55638 (47.49) | 0.5491 |  | 26106 (72.12) | 26059 (71.99) | 0.0029 |  | |
| Mental status | Alert | 35651 (98.48) | 115072 (98.22) | 0.0213 | 0.0101 | 35651 (98.48) | 35716 (98.66) | -0.0147 | 0.3118 | |
|  | Drowsy | 396 (1.09) | 1451 (1.24) | -0.0139 |  | 396 (1.09) | 354 (0.98) | 0.0112 |  | |
|  | Stupor | 97 (0.27) | 394 (0.34) | -0.0132 |  | 97 (0.27) | 79 (0.22) | 0.0096 |  | |
|  | Semicoma | 40 (0.11) | 150 (0.13) | -0.0053 |  | 40 (0.11) | 34 (0.09) | 0.0050 |  | |
|  | Coma | 16 (0.04) | 86 (0.07) | -0.0139 |  | 16 (0.04) | 17 (0.05) | -0.0013 |  | |
| Systolic blood pressure | -89 | 2593 (7.16) | 11927 (10.18) | -0.1170 | <0.0001 | 2593 (7.16) | 2553 (7.05) | 0.0043 | 0.3459 | |
|  | 90-139 | 21060 (58.18) | 65639 (56.03) | 0.0436 |  | 21060 (58.18) | 20917 (57.78) | 0.0080 |  | |
|  | 140- | 12547 (34.66) | 39587 (33.79) | 0.0183 |  | 12547 (34.66) | 12730 (35.17) | -0.0106 |  | |
| Pulse rate | -59 | 1096 (3.03) | 3569 (3.05) | -0.0011 | 0.0016 | 1096 (3.03) | 1051 (2.90) | 0.0073 | 0.3805 | |
|  | 60-99 | 26012 (71.86) | 85242 (72.76) | -0.0201 |  | 26012 (71.86) | 26159 (72.26) | -0.0090 |  | |
|  | 100- | 9092 (25.12) | 28342 (24.19) | 0.0213 |  | 9092 (25.12) | 8990 (24.83) | 0.0065 |  | |
| Respiratory rate | -11 | 144 (0.40) | 333 (0.28) | 0.0180 | 0.0031 | 144 (0.40) | 154 (0.43) | -0.0044 | 0.4220 | |
|  | 12-19 | 27774 (76.72) | 90069 (76.88) | -0.0037 |  | 27774 (76.72) | 27899 (77.07) | -0.0082 |  | |
|  | 20- | 8282 (22.88) | 26751 (22.83) | 0.0011 |  | 8282 (22.88) | 8147 (22.51) | 0.0089 |  | |
| Oxygen saturation | -89 | 407 (1.12) | 1089 (0.93) | 0.0185 | <0.0001 | 407 (1.12) | 405 (1.12) | 0.0005 | 0.0334 | |
|  | 90-94 | 1578 (4.36) | 4236 (3.62) | 0.0364 |  | 1578 (4.36) | 1438 (3.97) | 0.0189 |  | |
|  | 95- | 34215 (94.52) | 111828 (95.45) | -0.0412 |  | 34215 (94.52) | 34357 (94.91) | -0.0172 |  | |
| Body temperature | -35.9 | 1047 (2.89) | 4660 (3.98) | -0.0648 | <0.0001 | 1047 (2.89) | 963 (2.66) | 0.0138 | 0.0388 | |
|  | 36.0-37.9 | 30401 (83.98) | 93768 (80.04) | 0.1075 |  | 30401 (83.98) | 30627 (84.61) | -0.0170 |  | |
|  | 38.0- | 4752 (13.13) | 18725 (15.98) | -0.0846 |  | 4752 (13.13) | 4610 (12.74) | 0.0116 |  | |
| SMD, standardized mean difference; KTAS, Korean Triage and Acuity Scale; ENT, ear, nose, and throat | | | | | | | | | |  |
| a A value of SMD less than 0.1 indicates satisfactory balance of covariates between exposed and unexposed subjects. | | | | | | | | | |  |
| b All variables are expressed as count and (%). | | | | | | | | | |  |
