# Supplementary material for: Impact of emergency department overcrowding on the occurrence of in-hospital cardiac arrest
Source: PLoS One. 2025 Jan 17;20(1):e0317457. doi: 10.1371/journal.pone.0317457 (PMC11741635; doi:10.1371/journal.pone.0317457)
Supplement: S2 Table — (DOCX) [file pone.0317457.s002.docx]

| **S2 Table. Characteristics of patients in the full study cohort and the propensity score-matched cohort, stratified by emergency department overcrowding, based on the number of total occupying patients above 90%** | | | | | | | | | | |
| --- | --- | --- | --- | --- | --- | --- | --- | --- | --- | --- |
| **Variables** | | **Full-study cohort** | | | | **Propensity score-matched cohort** | | | | |
|  |  | Overcrowding (n = 17111) | Non-overcrowding (n = 136242) | SMD | p-value | Overcrowding (n = 17110) | Non-overcrowding (n = 17110) | SMD | p-value | |
| Age | -39 | 4637 (27.10) | 45897 (33.69) | -0.1482 | <0.0001 | 4637 (27.10) | 4667 (27.28) | -0.0039 | 0.4472 | |
|  | 40-64 | 6323 (36.95) | 48560 (35.64) | 0.0271 |  | 6323 (36.96) | 6389 (37.34) | -0.0080 |  | |
|  | 65-79 | 4508 (26.35) | 30613 (22.47) | 0.0880 |  | 4507 (26.34) | 4495 (26.27) | 0.0016 |  | |
|  | 80- | 1643 (9.60) | 11172 (8.20) | 0.0476 |  | 1643 (9.60) | 1559 (9.11) | 0.0167 |  | |
| Male |  | 8066 (47.14) | 63130 (46.34) | 0.0161 | 0.0472 | 8066 (47.14) | 8095 (47.31) | -0.0034 | 0.7535 | |
| Emergency medical services |  | 3627 (21.20) | 33902 (24.88) | -0.0902 | <0.0001 | 3627 (21.20) | 3457 (20.21) | 0.0243 | 0.0233 | |
| Transfer in |  | 3367 (19.68) | 15976 (11.73) | 0.2000 | <0.0001 | 3366 (19.67) | 3285 (19.20) | 0.0119 | 0.2685 | |
| KTAS | 1 | 178 (1.04) | 1463 (1.07) | -0.0033 | <0.0001 | 178 (1.04) | 177 (1.03) | 0.0006 | 0.7977 | |
|  | 2 | 1499 (8.76) | 11289 (8.29) | 0.0168 |  | 1499 (8.76) | 1447 (8.46) | 0.0107 |  | |
|  | 3 | 4828 (28.22) | 33873 (24.86) | 0.0745 |  | 4827 (28.21) | 4784 (27.96) | 0.0056 |  | |
|  | 4 | 8726 (51.00) | 71363 (52.38) | -0.0277 |  | 8726 (51.00) | 8824 (51.57) | -0.0115 |  | |
|  | 5 | 1880 (10.99) | 18254 (13.40) | -0.0771 |  | 1880 (10.99) | 1878 (10.98) | 0.0004 |  | |
| Non-medical |  | 2418 (14.13) | 24442 (17.94) | -0.1093 | <0.0001 | 2418 (14.13) | 2475 (14.47) | -0.0096 | 0.3787 | |
| Chief complaints | Gastrointestinal | 3426 (20.02) | 27510 (20.19) | -0.0042 | <0.0001 | 3426 (20.02) | 3461 (20.23) | -0.0051 | 0.9833 | |
|  | General | 2963 (17.32) | 22051 (16.19) | 0.0299 |  | 2963 (17.32) | 2892 (16.90) | 0.0110 |  | |
|  | Neurological | 2639 (15.42) | 19795 (14.53) | 0.0247 |  | 2639 (15.42) | 2654 (15.51) | -0.0024 |  | |
|  | Cardiovascular | 1851 (10.82) | 13129 (9.64) | 0.0380 |  | 1851 (10.82) | 1881 (10.99) | -0.0056 |  | |
|  | Musculoskeletal | 1526 (8.92) | 12689 (9.31) | -0.0139 |  | 1526 (8.92) | 1536 (8.98) | -0.0021 |  | |
|  | Respiratory | 1638 (9.57) | 9680 (7.11) | 0.0839 |  | 1637 (9.57) | 1597 (9.33) | 0.0079 |  | |
|  | Skin | 883 (5.16) | 9689 (7.11) | -0.0882 |  | 883 (5.16) | 889 (5.20) | -0.0016 |  | |
|  | ENT | 768 (4.49) | 8561 (6.28) | -0.0867 |  | 768 (4.49) | 774 (4.52) | -0.0017 |  | |
|  | Others | 1417 (8.28) | 13138 (9.64) | -0.0494 |  | 1417 (8.28) | 1426 (8.33) | -0.0019 |  | |
| Severe disease |  | 2238 (13.08) | 14794 (10.86) | 0.0659 | <0.0001 | 2237 (13.07) | 2126 (12.43) | 0.0192 | 0.0720 | |
| Area | Monitoring area | 1552 (9.07) | 10599 (7.78) | 0.0449 | <0.0001 | 1552 (9.07) | 1447 (8.46) | 0.0214 | 0.2475 | |
|  | Bed area | 2349 (13.73) | 26208 (19.24) | -0.1601 |  | 2349 (13.73) | 2360 (13.79) | -0.0019 |  | |
|  | Chair area | 445 (2.60) | 30456 (22.35) | -1.2412 |  | 445 (2.60) | 439 (2.57) | 0.0022 |  | |
|  | Fast track | 12765 (74.60) | 68979 (50.63) | 0.5507 |  | 12764 (74.60) | 12864 (75.18) | -0.0134 |  | |
| Mental status | Alert | 16871 (98.60) | 133852 (98.25) | 0.0299 | 0.0160 | 16870 (98.60) | 16909 (98.83) | -0.0194 | 0.4324 | |
|  | Drowsy | 170 (0.99) | 1677 (1.23) | -0.0239 |  | 170 (0.99) | 141 (0.82) | 0.0171 |  | |
|  | Stupor | 48 (0.28) | 443 (0.33) | -0.0084 |  | 48 (0.28) | 40 (0.23) | 0.0088 |  | |
|  | Semicoma | 15 (0.09) | 175 (0.13) | -0.0138 |  | 15 (0.09) | 15 (0.09) | 0.0000 |  | |
|  | Coma | 7 (0.04) | 95 (0.07) | -0.0143 |  | 7 (0.04) | 5 (0.03) | 0.0058 |  | |
| Systolic blood pressure | -89 | 893 (5.22) | 13627 (10.00) | -0.2151 | <0.0001 | 893 (5.22) | 851 (4.97) | 0.0110 | 0.5260 | |
|  | 90-139 | 10039 (58.67) | 76660 (56.27) | 0.0488 |  | 10038 (58.67) | 10105 (59.06) | -0.0080 |  | |
|  | 140- | 6179 (36.11) | 45955 (33.73) | 0.0496 |  | 6179 (36.11) | 6154 (35.97) | 0.0030 |  | |
| Pulse rate | -59 | 526 (3.07) | 4139 (3.04) | 0.0021 | 0.0003 | 526 (3.07) | 477 (2.79) | 0.0166 | 0.1383 | |
|  | 60-99 | 12196 (71.28) | 99058 (72.71) | -0.0316 |  | 12196 (71.28) | 12330 (72.06) | -0.0173 |  | |
|  | 100- | 4389 (25.65) | 33045 (24.25) | 0.0320 |  | 4388 (25.65) | 4303 (25.15) | 0.0114 |  | |
| Respiratory rate | -11 | 64 (0.37) | 413 (0.30) | 0.0116 | 0.1183 | 64 (0.37) | 52 (0.30) | 0.0115 | 0.2473 | |
|  | 12-19 | 13071 (76.39) | 104772 (76.90) | -0.0121 |  | 13070 (76.39) | 13176 (77.01) | -0.0146 |  | |
|  | 20- | 3976 (23.24) | 31057 (22.80) | 0.0104 |  | 3976 (23.24) | 3882 (22.69) | 0.0130 |  | |
| Oxygen saturation | -89 | 183 (1.07) | 1313 (0.96) | 0.0103 | <0.0001 | 183 (1.07) | 167 (0.98) | 0.0091 | 0.0960 | |
|  | 90-94 | 777 (4.54) | 5037 (3.70) | 0.0405 |  | 776 (4.54) | 702 (4.10) | 0.0208 |  | |
|  | 95- | 16151 (94.39) | 129892 (95.34) | -0.0413 |  | 16151 (94.40) | 16241 (94.92) | -0.0229 |  | |
| Body temperature | -35.9 | 512 (2.99) | 5195 (3.81) | -0.0482 | <0.0001 | 512 (2.99) | 470 (2.75) | 0.0144 | 0.0672 | |
|  | 36.0-37.9 | 14714 (85.99) | 109455 (80.34) | 0.1629 |  | 14713 (85.99) | 14858 (86.84) | -0.0244 |  | |
|  | 38.0- | 1885 (11.02) | 21592 (15.85) | -0.1543 |  | 1885 (11.02) | 1782 (10.42) | 0.0192 |  | |
| SMD, standardized mean difference; KTAS, Korean Triage and Acuity Scale; ENT, ear, nose, and throat | | | | | | | | | |  |
| a A value of SMD less than 0.1 indicates satisfactory balance of covariates between exposed and unexposed subjects. | | | | | | | | | |  |
| b All variables are expressed as count and (%). | | | | | | | | | |  |
