# Supplementary material for: Impact of emergency department overcrowding on the occurrence of in-hospital cardiac arrest
Source: PLoS One. 2025 Jan 17;20(1):e0317457. doi: 10.1371/journal.pone.0317457 (PMC11741635; doi:10.1371/journal.pone.0317457)
Supplement: S3 Table — (DOCX) [file pone.0317457.s003.docx]

| **S3 Table. Characteristics of patients in the full study cohort and the propensity score-matched cohort, stratified by emergency department overcrowding, based on the number of treating patients above the third quartile** | | | | | | | | | | |
| --- | --- | --- | --- | --- | --- | --- | --- | --- | --- | --- |
| **Variables** | | **Full-study cohort** | | | | **Propensity score-matched cohort** | | | | |
|  |  | Overcrowding (n = 52968) | Non-overcrowding (n = 100385) | SMD | p-value | Overcrowding (n = 52968) | Non-overcrowding (n = 52968) | SMD | p-value | |
| Age | -39 | 17265 (32.60) | 33269 (33.14) | -0.0117 | 0.0864 | 17265 (32.60) | 17334 (32.73) | -0.0028 | 0.1740 | |
|  | 40-64 | 19066 (36.00) | 35817 (35.68) | 0.0066 |  | 19066 (36.00) | 19195 (36.24) | -0.0051 |  | |
|  | 65-79 | 12126 (22.89) | 22995 (22.91) | -0.0003 |  | 12126 (22.89) | 12125 (22.89) | 0.0000 |  | |
|  | 80- | 4511 (8.52) | 8304 (8.27) | 0.0088 |  | 4511 (8.52) | 4314 (8.15) | 0.0133 |  | |
| Male |  | 24322 (45.92) | 46874 (46.69) | -0.0156 | 0.0038 | 24322 (45.92) | 24199 (45.69) | 0.0047 | 0.4482 | |
| Emergency medical services |  | 11735 (22.15) | 25794 (25.70) | -0.0852 | <0.0001 | 11735 (22.16) | 11302 (21.34) | 0.0197 | 0.0013 | |
| Transfer in |  | 8041 (15.18) | 11302 (11.26) | 0.1093 | <0.0001 | 8041 (15.18) | 7607 (14.36) | 0.0228 | 0.0002 | |
| KTAS | 1 | 593 (1.12) | 1048 (1.04) | 0.0072 | <0.0001 | 593 (1.12) | 555 (1.05) | 0.0068 | 0.2653 | |
|  | 2 | 4395 (8.30) | 8393 (8.36) | -0.0023 |  | 4395 (8.30) | 4238 (8.00) | 0.0107 |  | |
|  | 3 | 13623 (25.72) | 25078 (24.98) | 0.0169 |  | 13623 (25.72) | 13646 (25.76) | -0.0010 |  | |
|  | 4 | 27179 (51.31) | 52910 (52.71) | -0.0279 |  | 27179 (51.31) | 27228 (51.41) | -0.0019 |  | |
|  | 5 | 7178 (13.55) | 12956 (12.91) | 0.0189 |  | 7178 (13.55) | 7301 (13.78) | -0.0068 |  | |
| Non-medical |  | 9306 (17.57) | 17554 (17.49) | 0.0022 | 0.6864 | 9306 (17.57) | 9450 (17.84) | -0.0071 | 0.2464 | |
| Chief complaints | Gastrointestinal | 10054 (18.98) | 20882 (20.80) | -0.0464 | <0.0001 | 10054 (18.98) | 10089 (19.05) | -0.0017 | 0.7981 | |
|  | General | 9166 (17.30) | 15848 (15.79) | 0.0401 |  | 9166 (17.31) | 9040 (17.07) | 0.0063 |  | |
|  | Neurological | 7759 (14.65) | 14675 (14.62) | 0.0008 |  | 7759 (14.65) | 7739 (14.61) | 0.0011 |  | |
|  | Cardiovascular | 5269 (9.95) | 9711 (9.67) | 0.0091 |  | 5269 (9.95) | 5311 (10.03) | -0.0026 |  | |
|  | Musculoskeletal | 5169 (9.76) | 9046 (9.01) | 0.0252 |  | 5169 (9.76) | 5262 (9.93) | -0.0059 |  | |
|  | Respiratory | 4147 (7.83) | 7171 (7.14) | 0.0255 |  | 4147 (7.83) | 4017 (7.58) | 0.0091 |  | |
|  | Skin | 3568 (6.74) | 7004 (6.98) | -0.0096 |  | 3568 (6.74) | 3606 (6.81) | -0.0029 |  | |
|  | ENT | 3034 (5.73) | 6295 (6.27) | -0.0234 |  | 3034 (5.73) | 3079 (5.81) | -0.0037 |  | |
|  | Others | 4802 (9.07) | 9753 (9.72) | -0.0226 |  | 4802 (9.07) | 4825 (9.11) | -0.0015 |  | |
| Severe disease |  | 6027 (11.38) | 11005 (10.96) | 0.0131 | 0.0137 | 6027 (11.38) | 5755 (10.87) | 0.0162 | 0.0079 | |
| Area | Monitoring area | 4207 (7.94) | 7944 (7.91) | 0.0011 | <0.0001 | 4207 (7.94) | 4014 (7.58) | 0.0135 | 0.1078 | |
|  | Bed area | 9592 (18.11) | 18965 (18.89) | -0.0203 |  | 9592 (18.11) | 9520 (17.97) | 0.0035 |  | |
|  | Chair area | 6015 (11.36) | 24886 (24.79) | -0.4234 |  | 6015 (11.36) | 5993 (11.31) | 0.0013 |  | |
|  | Fast track | 33154 (62.59) | 48590 (48.40) | 0.2932 |  | 33154 (62.59) | 33441 (63.13) | -0.0112 |  | |
| Mental status | Alert | 52137 (98.43) | 98586 (98.21) | 0.0180 | 0.0304 | 52137 (98.43) | 52235 (98.62) | -0.0149 | 0.1466 | |
|  | Drowsy | 588 (1.11) | 1259 (1.25) | -0.0138 |  | 588 (1.11) | 510 (0.96) | 0.0141 |  | |
|  | Stupor | 151 (0.29) | 340 (0.34) | -0.0101 |  | 151 (0.29) | 134 (0.25) | 0.0060 |  | |
|  | Semicoma | 58 (0.11) | 132 (0.13) | -0.0067 |  | 58 (0.11) | 54 (0.10) | 0.0023 |  | |
|  | Coma | 34 (0.06) | 68 (0.07) | -0.0014 |  | 34 (0.06) | 35 (0.07) | -0.0007 |  | |
| Systolic blood pressure | -89 | 5004 (9.45) | 9516 (9.48) | -0.0011 | 0.8771 | 5004 (9.45) | 4720 (8.91) | 0.0183 | 0.0103 | |
|  | 90-139 | 29993 (56.62) | 56706 (56.49) | 0.0027 |  | 29993 (56.63) | 30185 (56.99) | -0.0073 |  | |
|  | 140- | 17971 (33.93) | 34163 (34.03) | -0.0022 |  | 17971 (33.93) | 18063 (34.10) | -0.0037 |  | |
| Pulse rate | -59 | 1528 (2.88) | 3137 (3.12) | -0.0144 | 0.0332 | 1528 (2.89) | 1402 (2.65) | 0.0142 | 0.0079 | |
|  | 60-99 | 38476 (72.64) | 72778 (72.50) | 0.0032 |  | 38476 (72.64) | 38852 (73.35) | -0.0159 |  | |
|  | 100- | 12964 (24.48) | 24470 (24.38) | 0.0023 |  | 12964 (24.48) | 12714 (24.00) | 0.0110 |  | |
| Respiratory rate | -11 | 113 (0.21) | 364 (0.36) | -0.0324 | 0.0031 | 113 (0.21) | 91 (0.17) | 0.0090 | 0.0589 | |
|  | 12-19 | 41559 (78.46) | 76284 (75.99) | 0.0601 |  | 41559 (78.46) | 41817 (78.95) | -0.0118 |  | |
|  | 20- | 11296 (21.33) | 23737 (23.65) | -0.0566 |  | 11296 (21.33) | 11060 (20.88) | 0.0109 |  | |
| Oxygen saturation | -89 | 517 (0.98) | 979 (0.98) | 0.0001 | 0.0307 | 517 (0.98) | 481 (0.91) | 0.0069 | 0.0017 | |
|  | 90-94 | 2102 (3.97) | 3712 (3.70) | 0.0139 |  | 2102 (3.97) | 1894 (3.58) | 0.0201 |  | |
|  | 95- | 50349 (95.06) | 95694 (95.33) | -0.0125 |  | 50349 (95.06) | 50593 (95.52) | -0.0212 |  | |
| Body temperature | -35.9 | 1433 (2.71) | 4274 (4.26) | -0.0957 | <0.0001 | 1433 (2.71) | 1326 (2.50) | 0.0125 | 0.0001 | |
|  | 36.0-37.9 | 43207 (81.57) | 80962 (80.65) | 0.0237 |  | 43207 (81.57) | 43725 (82.55) | -0.0252 |  | |
|  | 38.0- | 8328 (15.72) | 15149 (15.09) | 0.0174 |  | 8328 (15.72) | 7917 (14.95) | 0.0213 |  | |
| SMD, standardized mean difference; KTAS, Korean Triage and Acuity Scale; ENT, ear, nose, and throat | | | | | | | | | |  |
| a A value of SMD less than 0.1 indicates satisfactory balance of covariates between exposed and unexposed subjects. | | | | | | | | | |  |
| b All variables are expressed as count and (%). | | | | | | | | | |  |
