# Supplementary material for: Impact of emergency department overcrowding on the occurrence of in-hospital cardiac arrest
Source: PLoS One. 2025 Jan 17;20(1):e0317457. doi: 10.1371/journal.pone.0317457 (PMC11741635; doi:10.1371/journal.pone.0317457)
Supplement: S4 Table — (DOCX) [file pone.0317457.s004.docx]

| **S4 Table. Characteristics of patients in the full study cohort and the propensity score-matched cohort, stratified by emergency department overcrowding, based on the number of treating patients above 80%** | | | | | | | | | | |
| --- | --- | --- | --- | --- | --- | --- | --- | --- | --- | --- |
| **Variables** | | **Full-study cohort** | | | | **Propensity score-matched cohort** | | | | |
|  |  | Overcrowding (n = 44169) | Non-overcrowding (n = 109184) | SMD | p-value | Overcrowding (n = 44169) | Non-overcrowding (n = 44169) | SMD | p-value | |
| Age | -39 | 14388 (32.57) | 36146 (33.11) | -0.0113 | 0.0919 | 14388 (32.58) | 14555 (32.95) | -0.0081 | 0.3442 | |
|  | 40-64 | 15974 (36.17) | 38909 (35.64) | 0.0110 |  | 15974 (36.17) | 15957 (36.13) | 0.0008 |  | |
|  | 65-79 | 10066 (22.79) | 25055 (22.95) | -0.0038 |  | 10066 (22.79) | 10047 (22.75) | 0.0010 |  | |
|  | 80- | 3741 (8.47) | 9074 (8.31) | 0.0057 |  | 3741 (8.47) | 3610 (8.17) | 0.0107 |  | |
| Male |  | 20264 (45.88) | 50932 (46.65) | -0.0154 | 0.0062 | 20264 (45.88) | 20221 (45.78) | 0.0020 | 0.7715 | |
| Emergency medical services |  | 9741 (22.05) | 27788 (25.45) | -0.0819 | <0.0001 | 9741 (22.05) | 9409 (21.30) | 0.0181 | 0.0067 | |
| Transfer in |  | 6857 (15.52) | 12486 (11.44) | 0.1129 | <0.0001 | 6857 (15.52) | 6461 (14.63) | 0.0248 | 0.0002 | |
| KTAS | 1 | 498 (1.13) | 1143 (1.05) | 0.0076 | 0.0003 | 498 (1.13) | 416 (0.94) | 0.0176 | 0.0127 | |
|  | 2 | 3639 (8.24) | 9149 (8.38) | -0.0051 |  | 3639 (8.24) | 3464 (7.84) | 0.0144 |  | |
|  | 3 | 11336 (25.67) | 27365 (25.06) | 0.0138 |  | 11336 (25.67) | 11381 (25.77) | -0.0023 |  | |
|  | 4 | 22727 (51.45) | 57362 (52.54) | -0.0217 |  | 22727 (51.46) | 22937 (51.93) | -0.0095 |  | |
|  | 5 | 5969 (13.51) | 14165 (12.97) | 0.0158 |  | 5969 (13.51) | 5971 (13.52) | -0.0001 |  | |
| Non-medical |  | 7750 (17.55) | 19110 (17.50) | 0.0011 | 0.8385 | 7750 (17.55) | 7830 (17.73) | -0.0048 | 0.4801 | |
| Chief complaints | Gastrointestinal | 8328 (18.85) | 22608 (20.71) | -0.0473 | <0.0001 | 8328 (18.86) | 8395 (19.01) | -0.0039 | 0.8939 | |
|  | General | 7681 (17.39) | 17333 (15.88) | 0.0400 |  | 7681 (17.39) | 7637 (17.30) | 0.0026 |  | |
|  | Neurological | 6447 (14.60) | 15987 (14.64) | -0.0013 |  | 6447 (14.60) | 6431 (14.56) | 0.0010 |  | |
|  | Cardiovascular | 4406 (9.98) | 10574 (9.68) | 0.0097 |  | 4406 (9.98) | 4411 (9.99) | -0.0004 |  | |
|  | Musculoskeletal | 4349 (9.85) | 9866 (9.04) | 0.0272 |  | 4349 (9.85) | 4349 (9.85) | 0.0000 |  | |
|  | Respiratory | 3446 (7.80) | 7872 (7.21) | 0.0221 |  | 3446 (7.80) | 3330 (7.54) | 0.0098 |  | |
|  | Skin | 2972 (6.73) | 7600 (6.96) | -0.0093 |  | 2972 (6.73) | 2956 (6.69) | 0.0014 |  | |
|  | ENT | 2488 (5.63) | 6841 (6.27) | -0.0274 |  | 2488 (5.63) | 2524 (5.71) | -0.0035 |  | |
|  | Others | 4052 (9.17) | 10503 (9.62) | -0.0154 |  | 4052 (9.17) | 4136 (9.36) | -0.0066 |  | |
| Severe disease |  | 5000 (11.32) | 12032 (11.02) | 0.0095 | 0.0902 | 5000 (11.32) | 4607 (10.43) | 0.0281 | <0.0001 | |
| Area | Monitoring area | 3460 (7.83) | 8691 (7.96) | -0.0047 | <0.0001 | 3460 (7.83) | 3253 (7.37) | 0.0174 | 0.0389 | |
|  | Bed area | 7973 (18.05) | 20584 (18.85) | -0.0208 |  | 7973 (18.05) | 7893 (17.87) | 0.0047 |  | |
|  | Chair area | 4666 (10.56) | 26235 (24.03) | -0.4380 |  | 4666 (10.56) | 4655 (10.54) | 0.0008 |  | |
|  | Fast track | 28070 (63.55) | 53674 (49.16) | 0.2990 |  | 28070 (63.55) | 28368 (64.23) | -0.0140 |  | |
| Mental status | Alert | 43491 (98.46) | 107232 (98.21) | 0.0206 | 0.0163 | 43491 (98.47) | 43619 (98.76) | -0.0236 | 0.0079 | |
|  | Drowsy | 481 (1.09) | 1366 (1.25) | -0.0156 |  | 481 (1.09) | 384 (0.87) | 0.0212 |  | |
|  | Stupor | 123 (0.28) | 368 (0.34) | -0.0111 |  | 123 (0.28) | 105 (0.24) | 0.0077 |  | |
|  | Semicoma | 48 (0.11) | 142 (0.13) | -0.0065 |  | 48 (0.11) | 41 (0.09) | 0.0048 |  | |
|  | Coma | 26 (0.06) | 76 (0.07) | -0.0044 |  | 26 (0.06) | 20 (0.05) | 0.0056 |  | |
| Systolic blood pressure | -89 | 4169 (9.44) | 10351 (9.48) | -0.0014 | 0.9223 | 4169 (9.44) | 3918 (8.87) | 0.0194 | 0.0137 | |
|  | 90-139 | 25005 (56.61) | 61694 (56.50) | 0.0022 |  | 25005 (56.61) | 25162 (56.97) | -0.0072 |  | |
|  | 140- | 14995 (33.95) | 37139 (34.02) | -0.0014 |  | 14995 (33.95) | 15089 (34.16) | -0.0045 |  | |
| Pulse rate | -59 | 1249 (2.83) | 3416 (3.13) | -0.0182 | 0.0074 | 1249 (2.83) | 1125 (2.55) | 0.0169 | 0.0045 | |
|  | 60-99 | 32085 (72.64) | 79169 (72.51) | 0.0030 |  | 32085 (72.64) | 32436 (73.44) | -0.0178 |  | |
|  | 100- | 10835 (24.53) | 26599 (24.36) | 0.0039 |  | 10835 (24.53) | 10608 (24.02) | 0.0119 |  | |
| Respiratory rate | -11 | 96 (0.22) | 381 (0.35) | -0.0283 | <0.0001 | 96 (0.22) | 86 (0.20) | 0.0049 | 0.0850 | |
|  | 12-19 | 34692 (78.54) | 83151 (76.16) | 0.0581 |  | 34692 (78.54) | 34953 (79.14) | -0.0144 |  | |
|  | 20- | 9381 (21.24) | 25652 (23.49) | -0.0551 |  | 9381 (21.24) | 9130 (20.67) | 0.0139 |  | |
| Oxygen saturation | -89 | 431 (0.98) | 1065 (0.98) | 0.0000 | 0.0638 | 431 (0.98) | 370 (0.84) | 0.0140 | <0.0001 | |
|  | 90-94 | 1754 (3.97) | 4060 (3.72) | 0.0129 |  | 1754 (3.97) | 1520 (3.44) | 0.0271 |  | |
|  | 95- | 41984 (95.05) | 104059 (95.31) | -0.0117 |  | 41984 (95.05) | 42279 (95.72) | -0.0308 |  | |
| Body temperature | -35.9 | 1159 (2.62) | 4548 (4.17) | -0.0964 | <0.0001 | 1159 (2.62) | 1047 (2.37) | 0.0159 | 0.0025 | |
|  | 36.0-37.9 | 36049 (81.62) | 88120 (80.71) | 0.0234 |  | 36049 (81.62) | 36409 (82.43) | -0.0210 |  | |
|  | 38.0- | 6961 (15.76) | 16516 (15.13) | 0.0174 |  | 6961 (15.76) | 6713 (15.20) | 0.0154 |  | |
| SMD, standardized mean difference; KTAS, Korean Triage and Acuity Scale; ENT, ear, nose, and throat | | | | | | | | | |  |
| a A value of SMD less than 0.1 indicates satisfactory balance of covariates between exposed and unexposed subjects. | | | | | | | | | |  |
| b All variables are expressed as count and (%). | | | | | | | | | |  |
