# Supplementary material for: Impact of emergency department overcrowding on the occurrence of in-hospital cardiac arrest
Source: PLoS One. 2025 Jan 17;20(1):e0317457. doi: 10.1371/journal.pone.0317457 (PMC11741635; doi:10.1371/journal.pone.0317457)
Supplement: S5 Table — (DOCX) [file pone.0317457.s005.docx]

| **S5 Table. Characteristics of patients in the full study cohort and the propensity score-matched cohort, stratified by emergency department overcrowding, based on the number of treating patients above 90%** | | | | | | | | | | |
| --- | --- | --- | --- | --- | --- | --- | --- | --- | --- | --- |
| **Variables** | | **Full-study cohort** | | | | **Propensity score-matched cohort** | | | | |
|  |  | Overcrowding (n = 22157) | Non-overcrowding (n = 131196) | SMD | p-value | Overcrowding (n = 22157) | Non-overcrowding (n = 22157) | SMD | p-value | |
| Age | -39 | 7064 (31.88) | 43470 (33.13) | -0.0269 | 0.0036 | 7064 (31.88) | 7237 (32.66) | -0.0168 | 0.0795 | |
|  | 40-64 | 8055 (36.35) | 46828 (35.69) | 0.0137 |  | 8055 (36.35) | 8061 (36.38) | -0.0006 |  | |
|  | 65-79 | 5147 (23.23) | 29974 (22.85) | 0.0091 |  | 5147 (23.23) | 5095 (23.00) | 0.0056 |  | |
|  | 80- | 1891 (8.53) | 10924 (8.33) | 0.0074 |  | 1891 (8.54) | 1764 (7.96) | 0.0205 |  | |
| Male |  | 10247 (46.25) | 60949 (46.46) | -0.0042 | 0.5636 | 10247 (46.25) | 10185 (45.97) | 0.0056 | 0.5546 | |
| Emergency medical services |  | 4939 (22.29) | 32590 (24.84) | -0.0613 | <0.0001 | 4939 (22.29) | 4633 (20.91) | 0.0332 | 0.0004 | |
| Transfer in |  | 3674 (16.58) | 15669 (11.94) | 0.1247 | <0.0001 | 3674 (16.58) | 3435 (15.50) | 0.0290 | 0.0020 | |
| KTAS | 1 | 255 (1.15) | 1386 (1.06) | 0.0089 | 0.0006 | 255 (1.15) | 200 (0.90) | 0.0233 | 0.0187 | |
|  | 2 | 1878 (8.48) | 10910 (8.32) | 0.0057 |  | 1878 (8.48) | 1770 (7.99) | 0.0175 |  | |
|  | 3 | 5808 (26.21) | 32893 (25.07) | 0.0260 |  | 5808 (26.21) | 5754 (25.97) | 0.0055 |  | |
|  | 4 | 11298 (50.99) | 68791 (52.43) | -0.0289 |  | 11298 (50.99) | 11491 (51.86) | -0.0174 |  | |
|  | 5 | 2918 (13.17) | 17216 (13.12) | 0.0014 |  | 2918 (13.17) | 2942 (13.28) | -0.0032 |  | |
| Non-medical |  | 3776 (17.04) | 23084 (17.60) | -0.0147 | 0.0452 | 3776 (17.04) | 3822 (17.25) | -0.0055 | 0.5621 | |
| Chief complaints | Gastrointestinal | 4180 (18.87) | 26756 (20.39) | -0.0391 | <0.0001 | 4180 (18.87) | 4232 (19.10) | -0.0060 | 0.7772 | |
|  | General | 3901 (17.61) | 21113 (16.09) | 0.0397 |  | 3901 (17.61) | 3913 (17.66) | -0.0014 |  | |
|  | Neurological | 3238 (14.61) | 19196 (14.63) | -0.0005 |  | 3238 (14.61) | 3187 (14.38) | 0.0065 |  | |
|  | Cardiovascular | 2249 (10.15) | 12731 (9.70) | 0.0148 |  | 2249 (10.15) | 2267 (10.23) | -0.0027 |  | |
|  | Musculoskeletal | 2133 (9.63) | 12082 (9.21) | 0.0142 |  | 2133 (9.63) | 2122 (9.58) | 0.0017 |  | |
|  | Respiratory | 1825 (8.24) | 9493 (7.24) | 0.0364 |  | 1825 (8.24) | 1719 (7.76) | 0.0174 |  | |
|  | Skin | 1403 (6.33) | 9169 (6.99) | -0.0270 |  | 1403 (6.33) | 1428 (6.45) | -0.0046 |  | |
|  | ENT | 1197 (5.40) | 8132 (6.20) | -0.0352 |  | 1197 (5.40) | 1223 (5.52) | -0.0052 |  | |
|  | Others | 2031 (9.17) | 12524 (9.55) | -0.0132 |  | 2031 (9.17) | 2066 (9.32) | -0.0055 |  | |
| Severe disease |  | 2598 (11.73) | 14434 (11.00) | 0.0225 | 0.0015 | 2598 (11.73) | 2385 (10.76) | 0.0299 | 0.0014 | |
| Area | Monitoring area | 1839 (8.30) | 10312 (7.86) | 0.0159 | <0.0001 | 1839 (8.30) | 1635 (7.38) | 0.0334 | 0.0021 | |
|  | Bed area | 4023 (18.16) | 24534 (18.70) | -0.0141 |  | 4023 (18.16) | 3971 (17.92) | 0.0061 |  | |
|  | Chair area | 1888 (8.52) | 29013 (22.11) | -0.4869 |  | 1888 (8.52) | 1881 (8.49) | 0.0011 |  | |
|  | Fast track | 14407 (65.02) | 67337 (51.33) | 0.2872 |  | 14407 (65.02) | 14670 (66.21) | -0.0249 |  | |
| Mental status | Alert | 21795 (98.37) | 128928 (98.27) | 0.0075 | 0.5782 | 21795 (98.37) | 21899 (98.84) | -0.0370 | 0.0010 | |
|  | Drowsy | 264 (1.19) | 1583 (1.21) | -0.0014 |  | 264 (1.19) | 190 (0.86) | 0.0308 |  | |
|  | Stupor | 61 (0.28) | 430 (0.33) | -0.0100 |  | 61 (0.28) | 45 (0.20) | 0.0138 |  | |
|  | Semicoma | 26 (0.12) | 164 (0.13) | -0.0022 |  | 26 (0.12) | 18 (0.08) | 0.0105 |  | |
|  | Coma | 11 (0.05) | 91 (0.07) | -0.0089 |  | 11 (0.05) | 5 (0.02) | 0.0122 |  | |
| Systolic blood pressure | -89 | 2039 (9.20) | 12481 (9.51) | -0.0107 | 0.0129 | 2039 (9.20) | 2011 (9.08) | 0.0044 | 0.8491 | |
|  | 90-139 | 12726 (57.44) | 73973 (56.38) | 0.0213 |  | 12726 (57.44) | 12711 (57.37) | 0.0014 |  | |
|  | 140- | 7392 (33.36) | 44742 (34.10) | -0.0157 |  | 7392 (33.36) | 7435 (33.56) | -0.0041 |  | |
| Pulse rate | -59 | 632 (2.85) | 4033 (3.07) | -0.0133 | 0.0518 | 632 (2.85) | 544 (2.46) | 0.0239 | 0.0177 | |
|  | 60-99 | 16008 (72.25) | 95246 (72.60) | -0.0078 |  | 16008 (72.25) | 16177 (73.01) | -0.0170 |  | |
|  | 100- | 5517 (24.9) | 31917 (24.33) | 0.0132 |  | 5517 (24.90) | 5436 (24.53) | 0.0085 |  | |
| Respiratory rate | -11 | 41 (0.19) | 436 (0.33) | -0.0343 | <0.0001 | 41 (0.19) | 31 (0.14) | 0.0105 | 0.0993 | |
|  | 12-19 | 17294 (78.05) | 100549 (76.64) | 0.0341 |  | 17294 (78.05) | 17457 (78.79) | -0.0178 |  | |
|  | 20- | 4822 (21.76) | 30211 (23.03) | -0.0306 |  | 4822 (21.76) | 4669 (21.07) | 0.0167 |  | |
| Oxygen saturation | -89 | 225 (1.02) | 1271 (0.97) | 0.0047 | <0.0001 | 225 (1.02) | 176 (0.79) | 0.0221 | <0.0001 | |
|  | 90-94 | 961 (4.34) | 4853 (3.70) | 0.0313 |  | 961 (4.34) | 790 (3.57) | 0.0379 |  | |
|  | 95- | 20971 (94.65) | 125072 (95.33) | -0.0304 |  | 20971 (94.65) | 21191 (95.64) | -0.0441 |  | |
| Body temperature | -35.9 | 580 (2.62) | 5127 (3.91) | -0.0808 | <0.0001 | 580 (2.62) | 489 (2.21) | 0.0257 | 0.0107 | |
|  | 36.0-37.9 | 18082 (81.61) | 106087 (80.86) | 0.0193 |  | 18082 (81.61) | 18240 (82.32) | -0.0184 |  | |
|  | 38.0- | 3495 (15.77) | 19982 (15.23) | 0.0149 |  | 3495 (15.77) | 3428 (15.47) | 0.0083 |  | |
| SMD, standardized mean difference; KTAS, Korean Triage and Acuity Scale; ENT, ear, nose, and throat | | | | | | | | | |  |
| a A value of SMD less than 0.1 indicates satisfactory balance of covariates between exposed and unexposed subjects. | | | | | | | | | |  |
| b All variables are expressed as count and (%). | | | | | | | | | |  |
