# Supplementary material for: Impact of emergency department overcrowding on the occurrence of in-hospital cardiac arrest
Source: PLoS One. 2025 Jan 17;20(1):e0317457. doi: 10.1371/journal.pone.0317457 (PMC11741635; doi:10.1371/journal.pone.0317457)
Supplement: S6 Table — (DOCX) [file pone.0317457.s006.docx]

| **S6 Table. Characteristics of patients in the full study cohort and the propensity score-matched cohort, stratified by emergency department overcrowding, based on the number of boarding patients above the third quartile** | | | | | | | | | | |
| --- | --- | --- | --- | --- | --- | --- | --- | --- | --- | --- |
| **Variables** | | **Full-study cohort** | | | | **Propensity score-matched cohort** | | | | |
|  |  | Overcrowding (n = 34535) | Non-overcrowding (n = 118818) | SMD | p-value | Overcrowding (n = 34530) | Non-overcrowding (n = 34530) | SMD | p-value | |
| Age | -39 | 9710 (28.12) | 40824 (34.36) | -0.1388 | <0.0001 | 9710 (28.12) | 9834 (28.48) | -0.0080 | 0.0969 | |
|  | 40-64 | 12464 (36.09) | 42419 (35.70) | 0.0081 |  | 12463 (36.09) | 12571 (36.41) | -0.0065 |  | |
|  | 65-79 | 9045 (26.19) | 26076 (21.95) | 0.0965 |  | 9041 (26.18) | 8987 (26.03) | 0.0036 |  | |
|  | 80- | 3316 (9.60) | 9499 (7.99) | 0.0546 |  | 3316 (9.60) | 3138 (9.09) | 0.0175 |  | |
| Male |  | 16201 (46.91) | 54995 (46.29) | 0.0126 | 0.0398 | 16198 (46.91) | 16192 (46.89) | 0.0003 | 0.9635 | |
| Emergency medical services |  | 8107 (23.47) | 29422 (24.76) | -0.0304 | <0.0001 | 8107 (23.48) | 7913 (22.92) | 0.0133 | 0.0803 | |
| Transfer in |  | 5669 (16.42) | 13674 (11.51) | 0.1325 | <0.0001 | 5665 (16.41) | 5679 (16.45) | -0.0011 | 0.8857 | |
| KTAS | 1 | 384 (1.11) | 1257 (1.06) | 0.0051 | <0.0001 | 384 (1.11) | 344 (1.00) | 0.0110 | 0.3914 | |
|  | 2 | 3061 (8.86) | 9727 (8.19) | 0.0238 |  | 3060 (8.86) | 2965 (8.59) | 0.0097 |  | |
|  | 3 | 9747 (28.22) | 28954 (24.37) | 0.0857 |  | 9746 (28.23) | 9762 (28.27) | -0.0010 |  | |
|  | 4 | 17622 (51.03) | 62467 (52.57) | -0.0310 |  | 17619 (51.03) | 17738 (51.37) | -0.0069 |  | |
|  | 5 | 3721 (10.77) | 16413 (13.81) | -0.0980 |  | 3721 (10.78) | 3721 (10.78) | 0.0000 |  | |
| Non-medical |  | 4874 (14.11) | 21986 (18.50) | -0.1261 | <0.0001 | 4874 (14.12) | 4910 (14.22) | -0.0030 | 0.6944 | |
| Chief complaints | Gastrointestinal | 7281 (21.08) | 23655 (19.91) | 0.0288 | <0.0001 | 7281 (21.09) | 7347 (21.28) | -0.0047 | 0.7130 | |
|  | General | 5780 (16.74) | 19234 (16.19) | 0.0147 |  | 5777 (16.73) | 5701 (16.51) | 0.0059 |  | |
|  | Neurological | 5318 (15.40) | 17116 (14.41) | 0.0275 |  | 5317 (15.40) | 5383 (15.59) | -0.0053 |  | |
|  | Cardiovascular | 3757 (10.88) | 11223 (9.45) | 0.0460 |  | 3756 (10.88) | 3846 (11.14) | -0.0084 |  | |
|  | Musculoskeletal | 2981 (8.63) | 11234 (9.45) | -0.0293 |  | 2981 (8.63) | 3009 (8.71) | -0.0029 |  | |
|  | Respiratory | 2981 (8.63) | 8337 (7.02) | 0.0575 |  | 2981 (8.63) | 2862 (8.29) | 0.0123 |  | |
|  | Skin | 1838 (5.32) | 8734 (7.35) | -0.0904 |  | 1838 (5.32) | 1812 (5.25) | 0.0034 |  | |
|  | ENT | 1623 (4.70) | 7706 (6.49) | -0.0844 |  | 1623 (4.70) | 1638 (4.74) | -0.0021 |  | |
|  | Others | 2976 (8.62) | 11579 (9.75) | -0.0402 |  | 2976 (8.620) | 2932 (8.49) | 0.0045 |  | |
| Severe disease |  | 4342 (12.57) | 12690 (10.68) | 0.0571 | <0.0001 | 4342 (12.58) | 4111 (11.91) | 0.0202 | 0.0073 | |
| Area | Monitoring area | 3073 (8.90) | 9078 (7.64) | 0.0442 | <0.0001 | 3073 (8.90) | 2884 (8.35) | 0.0192 | 0.0833 | |
|  | Bed area | 4791 (13.87) | 23766 (20.00) | -0.1773 |  | 4791 (13.88) | 4829 (13.99) | -0.0032 |  | |
|  | Chair area | 2829 (8.19) | 28072 (23.63) | -0.5628 |  | 2829 (8.19) | 2867 (8.30) | -0.0040 |  | |
|  | Fast track | 23842 (69.04) | 57902 (48.73) | 0.4392 |  | 23837 (69.03) | 23950 (69.36) | -0.0071 |  | |
| Mental status | Alert | 33977 (98.38) | 116746 (98.26) | 0.0102 | 0.3768 | 33972 (98.38) | 34030 (98.55) | -0.0133 | 0.3625 | |
|  | Drowsy | 381 (1.10) | 1466 (1.23) | -0.0125 |  | 381 (1.10) | 337 (0.98) | 0.0122 |  | |
|  | Stupor | 108 (0.31) | 383 (0.32) | -0.0017 |  | 108 (0.31) | 107 (0.31) | 0.0005 |  | |
|  | Semicoma | 46 (0.13) | 144 (0.12) | 0.0033 |  | 46 (0.13) | 35 (0.10) | 0.0087 |  | |
|  | Coma | 23 (0.07) | 79 (0.07) | 0.0000 |  | 23 (0.07) | 21 (0.06) | 0.0022 |  | |
| Systolic blood pressure | -89 | 2475 (7.17) | 12045 (10.14) | -0.1152 | <0.0001 | 2474 (7.17) | 2297 (6.65) | 0.0199 | 0.0288 | |
|  | 90-139 | 19899 (57.62) | 66800 (56.22) | 0.0283 |  | 19898 (57.63) | 20033 (58.02) | -0.0079 |  | |
|  | 140- | 12161 (35.21) | 39973 (33.64) | 0.0329 |  | 12158 (35.21) | 12200 (35.33) | -0.0025 |  | |
| Pulse rate | -59 | 1137 (3.29) | 3528 (2.97) | 0.0181 | <0.0001 | 1136 (3.29) | 1101 (3.19) | 0.0057 | 0.2552 | |
|  | 60-99 | 24777 (71.74) | 86477 (72.78) | -0.0230 |  | 24773 (71.74) | 24965 (72.30) | -0.0123 |  | |
|  | 100- | 8621 (24.96) | 28813 (24.25) | 0.0165 |  | 8621 (24.97) | 8464 (24.51) | 0.0105 |  | |
| Respiratory rate | -11 | 200 (0.58) | 277 (0.23) | 0.0456 | <0.0001 | 195 (0.57) | 179 (0.52) | 0.0061 | 0.4101 | |
|  | 12-19 | 25865 (74.9) | 91978 (77.41) | -0.0580 |  | 25865 (74.91) | 25995 (75.28) | -0.0087 |  | |
|  | 20- | 8470 (24.53) | 26563 (22.36) | 0.0504 |  | 8470 (24.53) | 8356 (24.20) | 0.0077 |  | |
| Oxygen saturation | -89 | 382 (1.11) | 1114 (0.94) | 0.0161 | <0.0001 | 381 (1.10) | 339 (0.98) | 0.0116 | 0.0025 | |
|  | 90-94 | 1498 (4.34) | 4316 (3.63) | 0.0346 |  | 1498 (4.34) | 1339 (3.88) | 0.0226 |  | |
|  | 95- | 32655 (94.56) | 113388 (95.43) | -0.0385 |  | 32651 (94.56) | 32852 (95.14) | -0.0257 |  | |
| Body temperature | -35.9 | 1300 (3.76) | 4407 (3.71) | 0.0029 | <0.0001 | 1299 (3.76) | 1234 (3.57) | 0.0099 | 0.1020 | |
|  | 36.0-37.9 | 28806 (83.41) | 95363 (80.26) | 0.0847 |  | 28802 (83.41) | 29005 (84.00) | -0.0158 |  | |
|  | 38.0- | 4429 (12.82) | 19048 (16.03) | -0.0959 |  | 4429 (12.83) | 4291 (12.43) | 0.0120 |  | |
| SMD, standardized mean difference; KTAS, Korean Triage and Acuity Scale; ENT, ear, nose, and throat | | | | | | | | | |  |
| a A value of SMD less than 0.1 indicates satisfactory balance of covariates between exposed and unexposed subjects. | | | | | | | | | |  |
| b All variables are expressed as count and (%). | | | | | | | | | |  |
