# Supplementary material for: Impact of emergency department overcrowding on the occurrence of in-hospital cardiac arrest
Source: PLoS One. 2025 Jan 17;20(1):e0317457. doi: 10.1371/journal.pone.0317457 (PMC11741635; doi:10.1371/journal.pone.0317457)
Supplement: S7 Table — (DOCX) [file pone.0317457.s007.docx]

| **S7 Table. Characteristics of patients in the full study cohort and the propensity score-matched cohort, stratified by emergency department overcrowding, based on the number of boarding patients above 80%** | | | | | | | | | | |
| --- | --- | --- | --- | --- | --- | --- | --- | --- | --- | --- |
| **Variables** | | **Full-study cohort** | | | | **Propensity score-matched cohort** | | | | |
|  |  | Overcrowding (n = 27336) | Non-overcrowding (n = 126017) | SMD | p-value | Overcrowding (n = 27334) | Non-overcrowding (n = 27334) | SMD | p-value | |
| Age | -39 | 7512 (27.48) | 43022 (34.14) | -0.1492 | <0.0001 | 7512 (27.48) | 7593 (27.78) | -0.0066 | 0.2858 | |
|  | 40-64 | 9878 (36.14) | 45005 (35.71) | 0.0088 |  | 9878 (36.14) | 9922 (36.30) | -0.0034 |  | |
|  | 65-79 | 7280 (26.63) | 27841 (22.09) | 0.1027 |  | 7279 (26.63) | 7284 (26.65) | -0.0004 |  | |
|  | 80- | 2666 (9.75) | 10149 (8.05) | 0.0573 |  | 2665 (9.75) | 2535 (9.27) | 0.0160 |  | |
| Male |  | 12856 (47.03) | 58340 (46.30) | 0.0147 | 0.0273 | 12856 (47.03) | 12981 (47.49) | -0.0092 | 0.2842 | |
| Emergency medical services |  | 6276 (22.96) | 31253 (24.80) | -0.0438 | <0.0001 | 6276 (22.96) | 6110 (22.35) | 0.0144 | 0.0899 | |
| Transfer in |  | 4532 (16.58) | 14811 (11.75) | 0.1298 | <0.0001 | 4531 (16.58) | 4567 (16.71) | -0.0035 | 0.6793 | |
| KTAS | 1 | 299 (1.09) | 1342 (1.06) | 0.0028 | <0.0001 | 299 (1.09) | 227 (0.83) | 0.0253 | 0.0225 | |
|  | 2 | 2420 (8.85) | 10368 (8.23) | 0.0220 |  | 2420 (8.85) | 2359 (8.63) | 0.0079 |  | |
|  | 3 | 7758 (28.38) | 30943 (24.55) | 0.0849 |  | 7756 (28.38) | 7846 (28.70) | -0.0073 |  | |
|  | 4 | 13931 (50.96) | 66158 (52.50) | -0.0307 |  | 13931 (50.97) | 13996 (51.20) | -0.0048 |  | |
|  | 5 | 2928 (10.71) | 17206 (13.65) | -0.0952 |  | 2928 (10.71) | 2906 (10.63) | 0.0026 |  | |
| Non-medical |  | 3747 (13.71) | 23113 (18.34) | -0.1347 | <0.0001 | 3747 (13.71) | 3756 (13.74) | -0.0010 | 0.9109 | |
| Chief complaints | Gastrointestinal | 5766 (21.09) | 25170 (19.97) | 0.0274 | <0.0001 | 5766 (21.10) | 5854 (21.42) | -0.0079 | 0.7282 | |
|  | General | 4529 (16.57) | 20485 (16.26) | 0.0084 |  | 4529 (16.57) | 4504 (16.48) | 0.0025 |  | |
|  | Neurological | 4251 (15.55) | 18183 (14.43) | 0.0310 |  | 4250 (15.55) | 4261 (15.59) | -0.0011 |  | |
|  | Cardiovascular | 3021 (11.05) | 11959 (9.49) | 0.0498 |  | 3021 (11.05) | 3110 (11.38) | -0.0104 |  | |
|  | Musculoskeletal | 2363 (8.64) | 11852 (9.41) | -0.0271 |  | 2363 (8.65) | 2351 (8.60) | 0.0016 |  | |
|  | Respiratory | 2419 (8.85) | 8899 (7.06) | 0.0629 |  | 2418 (8.85) | 2301 (8.42) | 0.0151 |  | |
|  | Skin | 1395 (5.10) | 9177 (7.28) | -0.0990 |  | 1395 (5.10) | 1401 (5.13) | -0.0010 |  | |
|  | ENT | 1255 (4.59) | 8074 (6.41) | -0.0868 |  | 1255 (4.59) | 1251 (4.58) | 0.0007 |  | |
|  | Others | 2337 (8.55) | 12218 (9.70) | -0.0410 |  | 2337 (8.55) | 2301 (8.42) | 0.0047 |  | |
| Severe disease |  | 3425 (12.53) | 13607 (10.80) | 0.0523 | <0.0001 | 3424 (12.53) | 3279 (12.00) | 0.0160 | 0.0587 | |
| Area | Monitoring area | 2485 (9.09) | 9666 (7.67) | 0.0494 | <0.0001 | 2485 (9.09) | 2307 (8.44) | 0.0227 | 0.0552 | |
|  | Bed area | 3553 (13.00) | 25004 (19.84) | -0.2035 |  | 3553 (13.00) | 3607 (13.20) | -0.0059 |  | |
|  | Chair area | 1780 (6.51) | 29121 (23.11) | -0.6727 |  | 1780 (6.51) | 1817 (6.65) | -0.0055 |  | |
|  | Fast track | 19518 (71.40) | 62226 (49.38) | 0.4873 |  | 19516 (71.40) | 19603 (71.72) | -0.0070 |  | |
| Mental status | Alert | 26917 (98.47) | 123806 (98.25) | 0.0180 | 0.1445 | 26915 (98.47) | 26988 (98.73) | -0.0217 | 0.0556 | |
|  | Drowsy | 290 (1.06) | 1557 (1.24) | -0.0170 |  | 290 (1.06) | 242 (0.89) | 0.0171 |  | |
|  | Stupor | 82 (0.30) | 409 (0.32) | -0.0045 |  | 82 (0.30) | 62 (0.23) | 0.0134 |  | |
|  | Semicoma | 31 (0.11) | 159 (0.13) | -0.0038 |  | 31 (0.11) | 33 (0.12) | -0.0022 |  | |
|  | Coma | 16 (0.06) | 86 (0.07) | -0.0040 |  | 16 (0.060) | 9 (0.03) | 0.0106 |  | |
| Systolic blood pressure | -89 | 1835 (6.71) | 12685 (10.07) | -0.1340 | <0.0001 | 1835 (6.71) | 1676 (6.13) | 0.0232 | 0.0209 | |
|  | 90-139 | 15785 (57.74) | 70914 (56.27) | 0.0298 |  | 15783 (57.74) | 15905 (58.19) | -0.0090 |  | |
|  | 140- | 9716 (35.54) | 42418 (33.66) | 0.0393 |  | 9716 (35.55) | 9753 (35.68) | -0.0028 |  | |
| Pulse rate | -59 | 892 (3.26) | 3773 (2.99) | 0.0151 | 0.0024 | 892 (3.26) | 824 (3.02) | 0.0140 | 0.0247 | |
|  | 60-99 | 19622 (71.78) | 91632 (72.71) | -0.0207 |  | 19621 (71.78) | 19887 (72.76) | -0.0216 |  | |
|  | 100- | 6822 (24.96) | 30612 (24.29) | 0.0153 |  | 6821 (24.95) | 6623 (24.23) | 0.0167 |  | |
| Respiratory rate | -11 | 155 (0.57) | 322 (0.26) | 0.0415 | <0.0001 | 154 (0.56) | 140 (0.51) | 0.0068 | 0.4171 | |
|  | 12-19 | 20620 (75.43) | 97223 (77.15) | -0.0399 |  | 20620 (75.44) | 20734 (75.85) | -0.0097 |  | |
|  | 20- | 6561 (24.00) | 28472 (22.59) | 0.0330 |  | 6560 (24.00) | 6460 (23.63) | 0.0086 |  | |
| Oxygen saturation | -89 | 303 (1.11) | 1193 (0.95) | 0.0154 | <0.0001 | 303 (1.110) | 260 (0.95) | 0.0150 | <0.0001 | |
|  | 90-94 | 1202 (4.40) | 4612 (3.66) | 0.0360 |  | 1201 (4.40) | 1015 (3.71) | 0.0332 |  | |
|  | 95- | 25831 (94.49) | 120212 (95.39) | -0.0394 |  | 25830 (94.50) | 26059 (95.34) | -0.0367 |  | |
| Body temperature | -35.9 | 1046 (3.83) | 4661 (3.70) | 0.0067 | <0.0001 | 1046 (3.83) | 999 (3.66) | 0.0090 | 0.2532 | |
|  | 36.0-37.9 | 22941 (83.92) | 101228 (80.33) | 0.0978 |  | 22939 (83.92) | 23077 (84.43) | -0.0137 |  | |
|  | 38.0- | 3349 (12.25) | 20128 (15.97) | -0.1135 |  | 3349 (12.25) | 3258 (11.92) | 0.0102 |  | |
| SMD, standardized mean difference; KTAS, Korean Triage and Acuity Scale; ENT, ear, nose, and throat | | | | | | | | | |  |
| a A value of SMD less than 0.1 indicates satisfactory balance of covariates between exposed and unexposed subjects. | | | | | | | | | |  |
| b All variables are expressed as count and (%). | | | | | | | | | |  |
