# Supplementary material for: Impact of emergency department overcrowding on the occurrence of in-hospital cardiac arrest
Source: PLoS One. 2025 Jan 17;20(1):e0317457. doi: 10.1371/journal.pone.0317457 (PMC11741635; doi:10.1371/journal.pone.0317457)
Supplement: S8 Table — (DOCX) [file pone.0317457.s008.docx]

| **S8 Table. Characteristics of patients in the full study cohort and the propensity score-matched cohort, stratified by emergency department overcrowding, based on the number of boarding patients above 90%** | | | | | | | | | | |
| --- | --- | --- | --- | --- | --- | --- | --- | --- | --- | --- |
| **Variables** | | **Full-study cohort** | | | | **Propensity score-matched cohort** | | | | |
|  |  | Overcrowding (n = 13201) | Non-overcrowding (n = 140152) | SMD | p-value | Overcrowding (n = 13201) | Non-overcrowding (n = 13201) | SMD | p-value | |
| Age | -39 | 3552 (26.91) | 46982 (33.52) | -0.1492 | <0.0001 | 3552 (26.91) | 3569 (27.04) | -0.0029 | 0.7082 | |
|  | 40-64 | 4827 (36.57) | 50056 (35.72) | 0.0176 |  | 4827 (36.57) | 4767 (36.11) | 0.0094 |  | |
|  | 65-79 | 3542 (26.83) | 31579 (22.53) | 0.0970 |  | 3542 (26.83) | 3612 (27.36) | -0.0120 |  | |
|  | 80- | 1280 (9.70) | 11535 (8.23) | 0.0495 |  | 1280 (9.70) | 1253 (9.49) | 0.0069 |  | |
| Male |  | 6205 (47.00) | 64991 (46.37) | 0.0127 | 0.1638 | 6205 (47.00) | 6213 (47.07) | -0.0012 | 0.9214 | |
| Emergency medical services |  | 2891 (21.90) | 34638 (24.71) | -0.0681 | <0.0001 | 2891 (21.90) | 2780 (21.06) | 0.0203 | 0.0962 | |
| Transfer in |  | 2227 (16.87) | 17116 (12.21) | 0.1244 | <0.0001 | 2227 (16.87) | 2190 (16.60) | 0.0075 | 0.5418 | |
| KTAS | 1 | 144 (1.09) | 1497 (1.07) | 0.0022 | <0.0001 | 144 (1.09) | 116 (0.88) | 0.0204 | 0.4916 | |
|  | 2 | 1138 (8.62) | 11650 (8.31) | 0.0110 |  | 1138 (8.62) | 1117 (8.46) | 0.0057 |  | |
|  | 3 | 3738 (28.32) | 34963 (24.95) | 0.0748 |  | 3738 (28.32) | 3731 (28.26) | 0.0012 |  | |
|  | 4 | 6742 (51.07) | 73347 (52.33) | -0.0252 |  | 6742 (51.07) | 6786 (51.41) | -0.0067 |  | |
|  | 5 | 1439 (10.90) | 18695 (13.34) | -0.0782 |  | 1439 (10.90) | 1451 (10.99) | -0.0029 |  | |
| Non-medical |  | 1765 (13.37) | 25095 (17.91) | -0.1333 | <0.0001 | 1765 (13.37) | 1766 (13.38) | -0.0002 | 0.9856 | |
| Chief complaints | Gastrointestinal | 2811 (21.29) | 28125 (20.07) | 0.0300 | <0.0001 | 2811 (21.29) | 2824 (21.39) | -0.0024 | 0.9266 | |
|  | General | 2148 (16.27) | 22866 (16.32) | -0.0012 |  | 2148 (16.27) | 2098 (15.89) | 0.0103 |  | |
|  | Neurological | 2111 (15.99) | 20323 (14.50) | 0.0407 |  | 2111 (15.99) | 2176 (16.48) | -0.0134 |  | |
|  | Cardiovascular | 1454 (11.01) | 13526 (9.65) | 0.0435 |  | 1454 (11.01) | 1470 (11.14) | -0.0039 |  | |
|  | Musculoskeletal | 1180 (8.94) | 13035 (9.30) | -0.0127 |  | 1180 (8.94) | 1195 (9.05) | -0.0040 |  | |
|  | Respiratory | 1209 (9.16) | 10109 (7.21) | 0.0675 |  | 1209 (9.16) | 1162 (8.80) | 0.0123 |  | |
|  | Skin | 610 (4.62) | 9962 (7.11) | -0.1185 |  | 610 (4.62) | 625 (4.73) | -0.0054 |  | |
|  | ENT | 584 (4.42) | 8745 (6.24) | -0.0883 |  | 584 (4.42) | 575 (4.36) | 0.0033 |  | |
|  | Others | 1094 (8.29) | 13461 (9.60) | -0.0478 |  | 1094 (8.29) | 1076 (8.15) | 0.0049 |  | |
| Severe disease |  | 1622 (12.29) | 15410 (11.00) | 0.0393 | <0.0001 | 1622 (12.29) | 1497 (11.34) | 0.0288 | 0.0172 | |
| Area | Monitoring area | 1244 (9.42) | 10907 (7.78) | 0.0562 | <0.0001 | 1244 (9.42) | 1122 (8.50) | 0.0316 | 0.0560 | |
|  | Bed area | 1493 (11.31) | 27064 (19.31) | -0.2526 |  | 1493 (11.31) | 1550 (11.74) | -0.0136 |  | |
|  | Chair area | 408 (3.09) | 30493 (21.76) | -1.0786 |  | 408 (3.09) | 411 (3.11) | -0.0013 |  | |
|  | Fast track | 10056 (76.18) | 71688 (51.15) | 0.5875 |  | 10056 (76.18) | 10118 (76.65) | -0.0110 |  | |
| Mental status | Alert | 13015 (98.59) | 137708 (98.26) | 0.0284 | 0.0553 | 13015 (98.59) | 13056 (98.90) | -0.0264 | 0.2429 | |
|  | Drowsy | 124 (0.94) | 1723 (1.23) | -0.0301 |  | 124 (0.94) | 96 (0.73) | 0.0220 |  | |
|  | Stupor | 39 (0.30) | 452 (0.32) | -0.0050 |  | 39 (0.30) | 29 (0.22) | 0.0140 |  | |
|  | Semicoma | 16 (0.12) | 174 (0.12) | -0.0008 |  | 16 (0.12) | 15 (0.11) | 0.0022 |  | |
|  | Coma | 7 (0.05) | 95 (0.07) | -0.0064 |  | 7 (0.05) | 5 (0.04) | 0.0066 |  | |
| Systolic blood pressure | -89 | 586 (4.44) | 13934 (9.94) | -0.2672 | <0.0001 | 586 (4.44) | 520 (3.94) | 0.0243 | 0.1271 | |
|  | 90-139 | 7733 (58.58) | 78966 (56.34) | 0.0454 |  | 7733 (58.58) | 7764 (58.81) | -0.0048 |  | |
|  | 140- | 4882 (36.98) | 47252 (33.71) | 0.0677 |  | 4882 (36.98) | 4917 (37.25) | -0.0055 |  | |
| Pulse rate | -59 | 422 (3.20) | 4243 (3.03) | 0.0096 | 0.1788 | 422 (3.20) | 385 (2.92) | 0.0159 | 0.3074 | |
|  | 60-99 | 9491 (71.90) | 101763 (72.61) | -0.0159 |  | 9491 (71.90) | 9573 (72.52) | -0.0138 |  | |
|  | 100- | 3288 (24.91) | 34146 (24.36) | 0.0126 |  | 3288 (24.91) | 3243 (24.57) | 0.0079 |  | |
| Respiratory rate | -11 | 82 (0.62) | 395 (0.28) | 0.0432 | <0.0001 | 82 (0.62) | 69 (0.52) | 0.0125 | 0.3396 | |
|  | 12-19 | 10118 (76.65) | 107725 (76.86) | -0.0051 |  | 10118 (76.65) | 10197 (77.24) | -0.0141 |  | |
|  | 20- | 3001 (22.73) | 32032 (22.86) | -0.0029 |  | 3001 (22.73) | 2935 (22.23) | 0.0119 |  | |
| Oxygen saturation | -89 | 148 (1.12) | 1348 (0.96) | 0.0151 | 0.0043 | 148 (1.12) | 129 (0.98) | 0.0137 | 0.0738 | |
|  | 90-94 | 558 (4.23) | 5256 (3.75) | 0.0237 |  | 558 (4.23) | 496 (3.76) | 0.0233 |  | |
|  | 95- | 12495 (94.65) | 133548 (95.29) | -0.0283 |  | 12495 (94.65) | 12576 (95.27) | -0.0273 |  | |
| Body temperature | -35.9 | 546 (4.14) | 5161 (3.68) | 0.0228 | <0.0001 | 546 (4.14) | 534 (4.05) | 0.0046 | 0.8096 | |
|  | 36.0-37.9 | 11404 (86.39) | 112765 (80.46) | 0.1729 |  | 11404 (86.39) | 11440 (86.66) | -0.0080 |  | |
|  | 38.0- | 1251 (9.48) | 22226 (15.86) | -0.2179 |  | 1251 (9.48) | 1227 (9.30) | 0.0062 |  | |
| SMD, standardized mean difference; KTAS, Korean Triage and Acuity Scale; ENT, ear, nose, and throat | | | | | | | | | |  |
| a A value of SMD less than 0.1 indicates satisfactory balance of covariates between exposed and unexposed subjects. | | | | | | | | | |  |
| b All variables are expressed as count and (%). | | | | | | | | | |  |
